# Marine Bioactive Components and Chronic Neuroinflammation: Focus on Neurodegenerative Disease

**DOI:** 10.3390/md23110446

**Published:** 2025-11-20

**Authors:** Elda Favari, Cinzia Parolini

**Affiliations:** 1 Department of Food and Drug, University of Parma, Parco Area delle Scienze 27/A, 43124 Parma, Italy; elda.favari@unipr.it; 2 Department of Pharmacological and Biomolecular Sciences, Rodolfo Paoletti, Università degli Studi di Milano, Via Balzaretti 9, 20133 Milano, Italy

**Keywords:** immune system, neurodegenerative diseases, neuroinflammation, Alzheimer’s disease, Parkinson’s disease, multiple sclerosis, amyotrophic lateral sclerosis, marine bioactive components, astaxanthin

## Abstract

Advances in neuroscience, immunology, and neuroimmunology have revealed that the nervous and immune systems form a bidirectional integrated network, ranging from regulating inflammation to directing stress responses, pivotal for the maintenance of the brain–body physiology. Like peripheral inflammation, neuroinflammation is a conserved process aimed at activating innate/adaptive immune and non-immune cells to effectively deal with bacteria, viruses, toxins, and injuries, and eventually at removing the microbial pathogens and supporting tissue repair and recovery. A failure of this process or the permanent release of pro-inflammatory mediators causes a condition called “chronic low-grade neuroinflammation” resulting in tissue damage and an increased risk of developing neurodegenerative diseases (NDD), such as Alzheimer’s disease (AD), Parkinson’s disease (PD), multiple sclerosis (MS), and amyotrophic lateral sclerosis (ALS). Marine-derived bioactive components are able to modulate lipid and glucose metabolism as well as inflammation and oxidative stress. In this review, we describe the neuroinflammatory process and its involvement in the pathogenesis and progression of AD, PD, MS, and ALS. Then, we discuss the potential therapeutic efficacy of select marine-derived bioactive components.

## 1. Introduction

The central nervous system (CNS) possesses a peculiar relationship with the immune system [1]. More connections between the nervous and immune systems have been recently identified as pivotal to brain–body physiology [2]. In the past, the term “immune privilege” was coined to indicate all rules that control neuroimmune interactions to protect the CNS from injuries while preventing collateral damage to neurons [3]. Moreover, the presence of the blood–brain barrier (BBB) and the absence of “classical” lymphatic vessels in the brain parenchyma prompted the notion of the immunological isolation of the CNS [4,5]. Therefore, researchers believed that neuroimmune interactions only occurred in pathological conditions where this isolation is disrupted.

It is well known that the CNS, being encased within the rigid borders of the skull and vertebral column, is peculiarly sensitive to edema resulting from inflammation, and neurons, primary cell components, are easily damaged and have limited capacity to be replaced if lost. Thus, the immune response must be strictly balanced to avoid damage and, on the other hand, to fight infections and help in tissue repair [1].

Advances in neuroscience, immunology, and neuroimmunology have revealed that the nervous and immune systems form a bidirectional integrated network, ranging from regulating inflammation to directing stress responses, rather than operating in isolation [2,6]. Indeed, Smyth et al., observing that the CNS is less “privileged” than originally thought, rejects tissue more slowly and is more tolerant than peripheral tissues, speculated the presence of a constellation of mechanisms that actively assure the immune status of the CNS [6]. In line with this hypothesis, Leunig et al. described the idea of the existence of a “sixth sense” to underscore the concept that the immune system recognizes what the nervous system cannot see, touch, smell, taste, or hear and the neuroimmune connectome highlights the deep interaction between these two systems [2]. This idea came from studies of the inflammatory reflex through which inflammatory cues trigger inflammatory responses via the vagus nerve. Of note, the nervous and immune systems act synergically and exchange information for the survival and health of the organism. Eventually, their interactions cause a broadly informed response to any stimulus [2]. Based on these premises, it is understandable that many neurodegenerative diseases (NDDs) comprise an immune component [7,8]. Specifically, in multiple sclerosis (MS), the immune system has been identified as the “driver” of this pathology, and in other NDDs, encompassing Alzheimer’s disease (AD), Parkinson’s disease (PD), and amyotrophic lateral sclerosis (ALS), a secondary immune component involved in the amplification of the disease has been found [9].

Bioactive components derived from marine environments proved efficacious in modulating lipid and glucose metabolism as well as inflammation and oxidative stress [10,11,12]. Besides omega-3 fatty acids [13], marine organisms are a source of different chemical substances, such as proteins/peptides, carotenoids (astaxanthin), polysaccharides, and polyphenols, that have shown strong anti-inflammatory and anti-oxidative effects [14,15,16,17]. Therefore, the regular consumption of these compounds throughout one’s life could represent a useful tool to improve health outcomes.

In this review, we first describe the neuroinflammatory process and its involvement in the pathogenesis and progression of NDDs such as AD, PD, MS, and ALS. Second, we discuss the potential therapeutic efficacy of select marine-derived bioactive components in the prevention/treatment of these NDDs.

## 2. Neuroinflammation

### 2.1. Pathophysiology

Neuroinflammation is the name given to the physiological and highly coordinated response of the CNS to threats to its integrity caused by a variety of conditions, including pathogens and trauma [12]. Like peripheral inflammation, neuroinflammation is a conserved process aimed at activating innate/adaptive immune and non-immune cells to effectively deal with bacteria, viruses, toxins, and injuries, and eventually removing the microbial pathogens and supporting tissue repair and recovery [18]. Therefore, the immune system plays a seminal role in the maintenance of tissue homeostasis and response to infection and injury [7]. Microglia, a type of glial cell, are the major resident immune cells in the brain and in the spinal cord. They are specialized macrophages that perform vital housekeeping functions, such as waste disposal and neuronal pruning, as well as the secretion of anti-inflammatory and neurotrophic factors [19].

**Figure 1:**
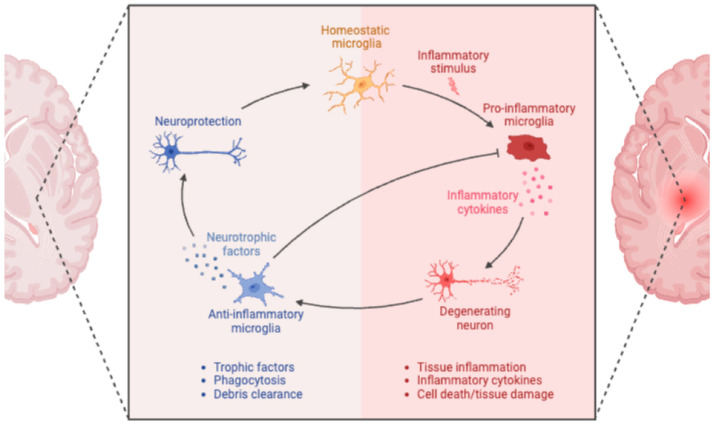
Roles of microglia in neuroinflammation. Created in BioRender. Cinzia Parolini (2025) https://app.biorender.com, assessed 25 September 2025.

Under physiological conditions, this highly regulated biological program comprises three different phases, namely, “onset phase”, “resolution phase”, and “adaptive homeostasis” [12]. Of note, in the “resolution phase”, different molecular and cellular events occur (1) the release of anti-inflammatory cytokines and specialized pro-resolving mediators (SPMs); (2) loss of receptors for pro-inflammatory stimuli; (3) activation of regulatory cells to dampen the activity of pro-inflammatory cells. Eventually, the aim is to restore tissue homeostasis [20]. A failure in this process or the permanent release of pro-inflammatory mediators causes a condition named “chronic low-grade neuroinflammation”, resulting in tissue damage, due to the production of neurotoxin factors that amply underlying disease states [21,22]. Of note, during these states, blood-borne pathogens can directly gain access to the CNS via the brain borders, namely the BBB and the blood–cerebrospinal fluid (CSF) barrier [23,24]. Microglia switch to an activated phenotype in response to infection or tissue damage and trigger an inflammatory reaction that serves to further engage the immune system and initiate tissue repair [19]. Therefore, genes exerting key roles in the amplification or effector functions of inflammatory responses are repressed under normal conditions and conversely are induced when cells recognize an infection or trauma (Figure 1).

Inflammatory responses to microbial pathogens begin when the pattern recognition receptors (PRRs) sense the microorganism motifs, named pathogen-associated molecular patterns (PAMPs) [25]. In addition, excessive inflammation triggers the release of damage-associated molecular patterns (DAMPs or alarmins [26], such as components released from necrotic cells and molecules produced through pathogenic mechanisms) that can bind and activate PRRs, causing exacerbated immune activation and organ dysfunction [27]. Lipopolysaccharides (LPS), peptidoglycan (PGN), lipoteichoic acid (LTA), bacterial lipoproteins, and nucleic acids are the most well-characterized PAMPs [28]. Four types of PRR families have been identified so far, namely Toll-like receptors (TLRs), C-type lectin receptors (CLRs), Retinoic acid-inducible gene (RIG)-like receptors (RLRs), and nucleotide-binding oligomerization domain (NOD)-like receptors (NLRs) [25]. These receptors are expressed by cells of innate immunity [that is, neutrophils, monocytes, dendritic cells (DCs), macrophages and microglia] [29] as well as fibroblasts, epithelial, and endothelial cells [7].

**Figure 2:**
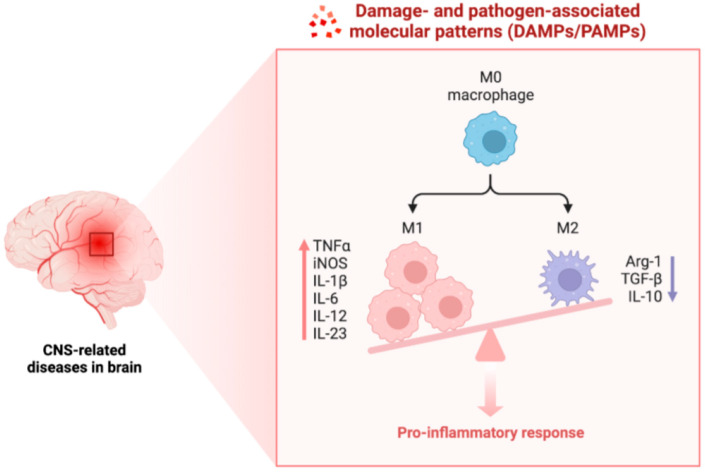
CNS-inflammatory responses to pathogens mediated by damage and pathogen-associated molecular patterns (DAMPs/PAMPs). Please refer to the text for the definitions of all abbreviations used in the figure. Created in BioRender. Cinzia Parolini (2025) https://app.biorender.com, assessed 25 September 2025.

Once activated, these PRRs lead to the up-regulation of the synthesis of genes coding for pro-inflammatory cytokines/chemokines as well as the activation of adaptive immunity and cellular metabolism. For example, TLRs signal both MyD88- (Myeloid differentiation primary response 88) and TRIF-dependent pathways, resulting in the phosphorylation/activation of the transcription factors, the inhibitory subunit beta of nuclear factor kappa B (NF-κB) kinase (IkB), three mitogen-activated protein kinases [MAPKs, namely ERK, p38, and c-Jun-N-terminal kinase (JNK)], and interferon regulatory factor 3 (IRF3) [25]. Eventually, these activated transcription factors migrate to the nucleus, bind to response elements, and mediate the gene expression of pro-inflammatory cytokines [such as interleukin (IL)-1, IL-6, IL-8, tumor necrosis factor (TNF)], chemokines [i.e., monocyte chemoattractant protein-1 (MCP-1)], antimicrobial peptides [e.g., inducible nitric oxide synthase (iNOS)], Type I interferons (IFNs), and IFN-inducible genes (Figure 2) [26].

The generation of reactive oxygen species (ROS) is also a fundamental antimicrobial system but may also represent a system causing collateral damage to tissue, such as the parenchymal cells in the brain. In addition, microglia also express purinergic and scavenger receptors which can be activated by the ATP released from cells and engage in the uptake of oxidized proteins, lipids, and apoptotic cells, and eventually contribute to inflammatory cell signaling [7]. Of note, knowing that TLR activation is a double-edged sword, it is understandable that these signaling pathways are strictly controlled by numerous negative feedback mechanisms, for example, soluble TLRs, SOCS proteins, A20, TOLLIP, ATF3, and phosphatidylinositol 3-kinases (PI3Ks) [25]. PI3Ks stimulate the synthesis of iNOS, vascular endothelial growth factor (VEGF), erythropoietin (EPO), glycolytic enzymes, IL-10, and diverse hypoxia-inducible factor (HIF)-target genes which enhance cell survival [30,31].

### 2.2. Neuroinflammation and Neurodegenerative Disease

Studies have highlighted the involvement of both innate and adaptive immune responses, triggered by chronic neuroinflammation, in the pathogenesis of NDD, mainly AD, PD, MS, and ALS. In detail, these data suggest that the neuropathological hallmarks of these disorders interacting with microglia and astrocytes stimulate an immune response and release of inflammatory mediators, eventually causing aggravation and progression of diseases [32,33,34].

#### 2.2.1. Alzheimer’s Disease (AD)

AD is an age-related neurodegenerative disorder and the most common cause of dementia, clinically manifested by progressive memory loss and impairments in cognitive and behavioral abilities [35]. The most common form of AD, called sporadic or late-onset AD (LOAD), accounts for 90–95% of all AD cases and usually manifests after age 65, with a marked correlation with the apolipoprotein E4 (apoE4) isoform [36,37]. While, the early-onset AD form, named EOAD, develops before age 65, and is often associated with autosomal dominant mutations in amyloid precursor protein (APP), presenilin (PSEN)1, or PSEN2 genes. It represents 5–10% of AD cases characterized by a more aggressive disease course, with a higher amyloid beta (Abeta) burden and immune system activation compared to the LOAD [38,39]. The pathological hallmarks in both AD forms are an abnormal extracellular deposition of Abeta plaques (constituted by Abeta1-42 and/or Abeta1-40 peptides) and neurofibrillary tangle (NFT) aggregation within neurons, eventually triggering oxidative stress and neuroinflammation, leading to the impairment of synaptic transmission, neuronal death, and brain atrophy [40]. Abeta1-42 and/or Abeta1-40 peptides are generated from APP by beta and gamma-secretases. Note, PSENs are the catalytic subunits of the gamma-secretase [41].

Currently, the amyloid cascade hypothesis is the main causative explanation of AD [42]. However, the lack of correlation between amyloid deposition and the severity of cognitive deficits, and the failure of the newly developed monoclonal antibodies directed against Abeta [43,44] prompt additional mechanisms contribute to disease pathogenesis, such as NFTs, synaptic loss, and microglial activation [45]. Therefore, it has now been proposed that neuroinflammation may also play a key role in the disease onset and progression [46,47]. Abeta plaques activate microglia by signaling through TLRs, NLRs, and receptor for advanced glycation endproducts (RAGE) [7]. These activated pathways trigger the nuclear translocation of transcription factors, such as NF-kB and AP-1, which in turn up-regulate the gene expression of TNF, IL-1beta, IL-6, and the production of ROS and NO and prostaglandins (i.e., PGE2) [48,49]. These inflammatory stimuli act both on cholinergic neurons, inducing apoptosis, and on astrocytes, leading to a further activation of microglia. Indeed, apoptosis and necrosis of neurons release ATP, which further stimulates microglia through the purinergic P2X7 receptor [50]. Moreover, inflammatory mediators acting on neurons might increase the production of Abeta peptides, generating a vicious cycle that contributes to AD pathology [7]. Of note, microglia play also a protective role by mediating the clearance of Abeta peptides by apoE-dependent and apoE-independent mechanisms [51]. Studies suggest that the presence of the apoE4 isoform protein might impair the clearance of Abeta peptides, eventually contributing to AD [7]. Furthermore, in addition to cholinergic neurons being those primarily affected in AD, other neurons, namely glutamatergic and GABAnergic neurons, could also represent crucial targets in AD pathology [50]. Finally, it is important to underline that environmental factors may trigger inflammatory responses that in turn could contribute to AD development, such as traumatic injury, systemic infections, and diet. Specifically, evidence supports a causal link between high body mass index, high fasting plasma glucose, smoking, high intake of refined sugar, and AD development [52].

#### 2.2.2. Parkinson’s Disease (PD)

PD is a chronic and progressive neurodegenerative disease and the most common movement disorder, characterized by the degeneration of dopaminergic neurons in the substantia nigra and by alpha-synuclein (aSyn) containing inclusion bodies (Lewy bodies and Lewy neurites) in the surviving neurons [53]. This leads to the appearance of motor (bradykinesia, resting tremors, rigidity and postural instability) and non-motor (sleep disturbance, mood disorders, cognitive decline, pain, and gastrointestinal dysfunction) symptoms [54]. Of note, some of these non-motor symptoms may appear many years before the occurrence of the classical motor signs [55,56] and their presence in healthy individuals has been linked to an increased risk of developing PD [57,58]. Even though most forms of PD are sporadic, the identification of genetic factors, monogenic mutations, and risk allele variants provides insights into the molecular mechanisms involved. A recently published observational study indicates that a genetic contributor can be recognized in about 15% of people with PD [59]. Among these, we can find mutations in PD-associated genes or missense variants, which result in diverse effects ranging from being “fully penetrant” or conferring “medium or strong predisposition” [60]. These PD-associated genes, over 200 so far, may also interact with other risk factors, such as aging and environmental milieu, to prompt the development of the pathology [61].

The pathology derives from aggregation of misfolded aSyn fibrils which, being insoluble, are deposited in neurons, glia, and nerve fibers. This protein is abundant in the brain and the reasons why it forms insoluble fibrils are not completely elucidated yet [62,63]. These intracellular inclusions disrupt cellular function, leading to neuronal death, microglia activation, and an increase in astroglia and lymphocyte infiltration [64]. In addition, it has been demonstrated that these aSyn aggregates are excreted in the extracellular space, where they are captured by cell surface gangliosides and internalized, most likely via lipid rafts, by microglia [65]. Here, they may act through two mechanisms: (1) activating the TLR signaling pathway, similarly to viruses and toxins, resulting in the synthesis of pro-inflammatory cytokines, such as IL-1, IL-6 and TNF-alpha, and (2) promoting the activation of the NADPH oxidase and iNOS with the release of ROS and NO, respectively [7]. These factors negatively influence the viability of dopaminergic neurons and trigger the activation of astrocytes. Of note, dopaminergic neurons in the substantia nigra are particularly sensitive to oxidative stress. Therefore, it is understandable why these neurons are the ones mainly involved in this pathology [66]. Moreover, conditions involving peripheral inflammation, such as stroke, infections, myocardial infarction, mechanical injury, or social stress, trigger a sepsis-like cytokine storm with the release of IL-6 and TNF-alpha, eventually leading to monocyte recruitment to the brain, worsening the neuroinflammatory milieu. Meanwhile, T cells are also observed to enter the brain to curb inflammatory responses [2]. For example, after stroke, regulatory T cells amass in the brain and release IL-4 and amphiregulin, suppressing neurotoxin astrogliosis and supporting brain recovery [67]. Finally, the transcription factor nuclear receptor-related 1 (NURR1) is an endogenous negative regulator of the expression of NF-KB target genes, induced in a ligand-dependent manner in both microglia and astrocytes [68].

#### 2.2.3. Multiple Sclerosis (MD)

MS is an autoimmune disease characterized by inflammation, demyelination, and axon degeneration in the CNS. MS mainly affects young women (as the most common non-traumatic disorder) [34]. MS manifests with diverse phenotypes: (i) relapsing–remitting MS (RRMS), the most frequent, characterized by incidents of neurological dysfunction that spontaneously recover; (ii) secondary progressive MS (SPMS), the evolution of RRMS, where affected individuals progressively acquire irreversible disability; (iii) primary progressive MS (PPMS), less frequent, where patients manifest irreversible and progressive disability at onset [69].

Studies assessed that MS is the result of a complex interplay of dysregulated immunity, genetic susceptibility, and environmental elements [34]. Indeed, key roles are played by different subpopulation of B and T cells, monocytes, DCs, astrocytes, and microglia. In addition, viral and bacterial infections may strongly contribute to the beginning of MS because of the molecular mimicry phenomenon that can take place between regions of PAMPs and regions of the myelin proteins, i.e., myelin basic protein (MBP), wherein the adaptive immune system starts to target specific myelin components [70]. Furthermore, a secretion of pro-inflammatory cytokines, such as IL-1beta and transforming growth factor (TGF)-beta, is observed as a result of the activation of the transcription factors NF-kB and AP-1 by activated astrocytes and microglia [7]. These cytokines trigger the differentiation of naïve T cells into T helper (Th)17 [70]. Furthermore, both activated microglia and astrocytes produce IL-23 and osteopontin, which act on Th17 cells and stimulate their secretion of IL-17 and TNF-alpha, eventually causing damage to the myelin sheath that protects nerve axons [7]. This detrimental effect is also supported by ROS and NO, produced upon the activation of NADPH oxidase and iNOS, in activated astrocytes and microglia, and by the astrocyte secretion of B-cell activating factor (BAFF), a survival factor for autoreactive B cells, which promote their differentiation in plasma cells, resulting in production of antibodies against myelin [7]. Similarly to what was described for PD, negative regulatory mechanisms can protect the brain from these exacerbated inflammatory responses. In detail, TGF-beta synthesized by microglia promotes the differentiation of T regulatory (Treg) cells that inhibits the activity of Th17, halting the vicious cycle [71].

#### 2.2.4. Amyotrophic Lateral Sclerosis (ALS)

ALS is a progressive and rapidly fatal NDD affecting motor neurons in the brainstem, spinal cord, and motor cortex. From a clinical point of view, this pathology is characterized by fasciculation, muscle wasting and weakness, increased spasticity, and hyper-reflexia, eventually leading to respiratory complications and death within 3–5 years [72]. Although the preponderance (90%) of ALS cases is sporadic (sALS), several genes have been identified as responsible for genetic heritability (fALS, 10%), namely, superoxide dismutase 1 (SOD1), chromosome 9 open reading frame 72 (C9orf72), fused in sarcoma (FUS), and transactive response DNA-binding protein 43 (TARDBP) [73,74]. The common pathological sign is the presence of ubiquitin-immunoreactive cytoplasmatic inclusions (aberrant protein aggregates) in degenerating neurons associated with a strong inflammatory reaction. Indeed, key players in this pathology are astrocytes, oligodendrocytes, microglia, and diverse peripheral immune cells [74]. In detail, experimental studies using mouse models of ALS have highlighted that before the onset of disease microglia express anti-inflammatory factors, such as insulin growth factor (IGF1, a neurotrophic factor) arginase 1 and IL-10 [75,76,77]. After the manifestation of the symptoms, microglia switch to a pro-inflammatory phenotype characterized by the production of IL-1beta, IL-6, and TNF [78,79]. Toxic aggregates trigger inflammatory responses by microglia via TLR2 [80] and its co-receptor CD14 [81], eventually activating NF-kB and AP-1 and up-regulating the secretion of inflammatory cytokines [7] and apoptosis-triggering molecules, such as TNF and Fas ligand (FASL) [82]. Moreover, these inflammatory cytokines activate the astrocytes. Altogether, these activated glial cells support this inflammatory milieu by promoting the release of ROS and NO [83]. These data were confirmed in ALS patients undergoing PET using a ligand that binds to the 18 KDa translocator protein (TSPO) [84]. Indeed, the TSPO PET signal mirrors the glial density and, given that TSPO expression is increased in disease-associated microglia compared to normal microglia, the neurodegenerative environment [85,86]. Furthermore, in individuals affected by ALS, a higher number of monocytes and neutrophils has been detected, with a pro-inflammatory phenotype, which has been correlated with increased disease burden and disease progression rates [87]. In addition, the involvement of adaptive immunity has been demonstrated in human studies. These data revealed that high levels of CD4+ T cells were associated with poor survival, whereas high amount of Treg cells were indicative of better survival [88]. Lastly, dying motor neurons secrete ATP in the extracellular space, which can also support the activation of microglia by binding to the P2XT receptor expressed by glia [89].

## 3. Marine-Derived Bioactive Components and AD, PD, MD, and ALS

Marine ecosystems represent a prolific source of structurally diverse bioactive molecules with significant biomedical potential. Among them, peptides, glycoproteins, lipids, carotenoids, sulfated polysaccharides, and polyphenols have shown anti-inflammatory, antioxidant, and neuroprotective effects that intersect with the pathogenesis of AD, PD, MS, and ALS [16,17,90,91]. These disorders share common features such as chronic neuroinflammation, persistent oxidative stress, mitochondrial dysfunction, and impaired BBB integrity. Marine-derived compounds act on multiple targets, ranging from the suppression of pro-inflammatory signaling (NF-kB, MAPKs) to the activation of cytoprotective and pro-resolving pathways (Nrf2/HO-1, PPARgamma, SIRT1). However, despite strong preclinical evidence, clinical translation remains limited and requires critical evaluation [92].

### 3.1. Proteins, Peptides and Amino Acids

Marine-derived peptides have demonstrated the capacity to inhibit NF-κB nuclear translocation and MAPKs (p38, ERK, JNK), reducing the transcription of pro-inflammatory cytokines (IL-1β, IL-6, TNF-alpha) and enzymes such as iNOS and COX-2 [93].

Their antioxidant properties, mediated through direct ROS scavenging and metal chelation, provide an additional layer of neuroprotection. Taurine, a sulfur-containing amino acid abundant in marine organisms, stabilizes cellular membranes, buffers intracellular calcium, and enhances inhibitory neurotransmission, thus counteracting excitotoxicity [94]. Beyond suppressing inflammation, bioactive peptides modulate nuclear receptors such as PPARgamma and SIRT1, reprogramming microglia toward anti-inflammatory phenotypes and enhancing trophic support via BDNF and GDNF. These data have been obtained from in vitro experiments, and some have also been confirmed in in vivo models of NDD, as recently reviewed [16,17,90,91].

Krill are small crustaceans frequently found in polar seas, whose largest species is the Antarctic *Euphasia superba* (Table 1). Besides being a source of omega-3 fatty acids [95], krill are also a valuable source of peptides with different biological activities, such as prevention of endothelial dysfunction and atherosclerosis development, and inhibition of inflammatory cascade, through a rise in NO [96] and a down-regulation of genes involved in HIF-2alpha and NLRP3 signaling pathways, respectively [97,98]. Two studies have demonstrated that krill peptides improve scopolamine-induced memory impairment in an in vivo model, by reducing neuronal cell harm caused by oxidative stress associated with SOD activity and ROS levels [99,100]. Moreover, Yang et al. have identified unique structural characteristics and immunomodulatory effects of peptides from Antarctic *Euphasia superba* in immunosuppressed mice [101].

In line with these data, sea cucumber (*Stichopus japonicus*) peptides (SCP) attenuated memory damage in both mice [102,103] and rats [103] treated with scopolamine. The authors demonstrated that SCP act in a dose-dependent manner and through mechanisms involving a rise in acetylcholine content, a reduction in the enzyme activity of the acetylcholinesterase, and up-regulation of the long-term potentiation (LTP) pathway and unsaturated lipid levels [103]. Gong et al. demonstrated that sea cucumber hydrolysates (SCH), enzymatically obtained from proteins isolated from *Stichopus japonicus*, improved behavioral deficits and hippocampal pathology in D-galactose-induced C57BL/6J aging mice [104]. These effects were mechanistically linked to neuroinflammatory suppression, through the up-regulation of BDNF/TrkB and the inhibition of NF-kB signaling pathways [104]. In addition, SCH modulated gut microbiota, along with increased fecal short-chain fatty acids levels. Functional prediction revealed that the suppression of neuroinflammation could be correlated with the signal transduction modification caused by gut microbiota modulation [104]. These results corroborate the clinical data suggesting that patients with PD develop intestinal inflammation [128], and the key role played by the gut–brain axis in the NDD [129].

A multi-functional peptide (YIAEDAER) isolated and purified from the meat and visceral mass of a marine snail, *Neptunea arthritica cumingii*, showed neuroprotective effects in a PD-like pathology in zebrafish [105]. The authors demonstrated that this peptide exerts anti-PD activity mainly via suppressing locomotor impairment, ameliorating the degeneration of dopaminergic neurons, and inhibiting the loss of cerebral vessels [105].

These converging findings support the potential use of marine-derived peptides as dietary supplements aimed at preventing NDD. Hower, the clinical validation is still lacking.

### 3.2. Astaxanthin

Astaxanthin, a marine carotenoid, is present in microalgae, such as *Heamatococcus pluvialis*, shrimp, lobster, crustacean, krill, trout, salmon (Table 1) [16,96]. It acts both as a potent antioxidant and as a regulator of inflammatory signaling. Its conjugated double-bond structure enables efficient quenching of ROS within lipid membranes [16]. Of note, data from mouse models suggest that astaxanthin may enhance the anti-inflammatory and anti-atherosclerotic activities of marine-derived oils [130,131].

Alugoju et al. have recently summarized the protective molecular mechanisms of astaxanthin actions on diverse NDD [132]. Briefly, in a mouse model of spinal cord injury (SCI), astaxanthin had marked reduced post-SCI sensory-motor dysfunction, by inhibiting the NF-κB signaling pathway, thereby reducing pro-inflammatory cytokine release and boosting endogenous antioxidant defenses [106]. Moreover, astaxanthin attenuated AD-related complications and reversed Abeta-induced insulin resistance in hippocampal neurons, through the inhibition of GSK-3beta activity [107]. In the same mouse model of AD, Liu et al. demonstrated that astaxanthin attenuated cognitive deficits by reducing oxidative stress, via the SIRT1/PGC-1alpha pathway [108]. In PD mouse models, astaxanthin protects dopaminergic neurons and reduces a-Syn aggregation [133,134].

In a mouse model of MS, namely experimental autoimmune encephalomyelitis (EAE), the consumption of astaxanthin, extracted from *Heamatococcus pluvialis*, exerts protective effects on disease prevention/progression, through the reduction in inflammatory infiltrates in the spinal cord and brain, and the stimulation of Treg cell differentiation [109]. Furthermore, astaxanthin reduced demyelination and oligodendrocyte death in a rat model of MS [110].

Preliminary clinical data, though not disease-specific, indicate systemic antioxidant and anti-inflammatory activity in humans [135]. However, large randomized controlled trials in neurodegeneration are still lacking.

### 3.3. Polysaccharides

Marine macroalgae, known as seaweed, are plant-like organisms that contain diverse bioactive compounds, mainly sulfated polysaccharides [136]. They can be divided into three groups based on their colors: (1) brown algae, which are rich in fucoidan; (2) red algae, which are rich in ulvan; (3) green algae, which are rich in carrageenan [137]. Evidence from in vitro experiments reported that fucoidan exert a neuroprotective function on NDD and brain injury by inhibiting inflammation (TNF-alpha, IL-1beta, iNOS, NOX-1), oxidative stress (ROS, MDA, Caspase 3/8/9), apoptosis, mitochondrial dysfunction (SOD, GSH-PX, PGC-1alpha, Bcl2, Sirt-3) and microglia activation, and by promoting neurite growth and the cholinergic system [90,138,139]. These results have been at least in part confirmed in in vivo studies (Table 1). In detail, orally administrated fucoidan, extracted from *Undaria pinnatifida* and *Laminaria japonica*, ameliorated the learning and memory impairments in two different AD mouse models, such as Abeta- and D-galactose-treated mice [111,112]. Fucoidan was able to regulate the cholinergic system, reduce the oxidative stress (ROS and MDA) and inhibit the caspase and mitochondrial apoptosis pathways (SOD, GSH-PX, Sirt-3) [111,112]. Comparable results were obtained by Zhang et al. using a polysaccharide from *Pyropia haitanensis* in a Abeta-induced AD mouse model [113]. Moreover, a fucoidan-rich extract from *Ecklonia cava* improved trimethyltin (TMT)-induced cognitive dysfunction in mice by downregulating Abeta production and tau hyperphosphorylation [114]. These results were also confirmed in AD transgenic *Caenorhabditis elegans (C. elegans)* cultured with fucoidan extracted from brown algae [115].

In addition, fucoidan extracted from *Laminaria japonica* (1) improved mitochondrial dysfunction involving the PGC-1alpha/NRF2 pathway in a rat model of PD, namely Sprague Dawley rats treated with rotenone [116]; (2) showed antioxidant activity in mice treated with 1-methyl-4-phenyl-1,2,3,6 tetrahydropyridine (MPTP) [117]; and (3) inhibited oxidation and microglial activation in Sprague Dawley rats treated with 6-hydroxydopamine (6-OHDA) [118]. Furthermore, fucoidan obtained from brown algae and *Turbinaria decurrens* improved behavioral deficits and inhibited microglial activation in Sprague Dawley rats treated with LPS [119], and C56Bl6 mice treated with MPTP [120], respectively. Neuroprotective effects, through the improvement of mitochondrial dysfunction and motor deficits, prevention of neuronal apoptosis, and decrease in dopaminergic neuron loss, were observed in a MPTP-induced PD mice treated with fucoidan isolated from *Fucus vesiculosus* [121]. Finally, a recent study performed in rotenone-treated mice, demonstrated that fucoidan, extracted from *Laminaria japonica*, are able to protect dopamine neurons indirectly by modulating the gut microbiota [122]. Indeed, fucoidan, sulfated polysaccharides which carry negative charges, may act as prebiotics, leading to a recovery of the PD-associated dysbiosis by decreasing the permeability of the intestinal barrier and decreasing the number of pro-inflammatory cytokines into both the systemic circulation and brain [122]. These results are in line with the growing evidence suggesting a pivotal role of the gut microbiota and the gut microbiota–brain axis in the development of PD [128].

In EAE rats, fucoidan attenuated demyelination and suppressed autoreactive T cell response and inflammatory cytokine production [123]. Nowadays, no data are available on the effects of fucoidan on ALS. However, Hsieh et al. demonstrated that fucoidan exert auxiliary neuroprotective activity on ALS, by reducing the ROS levels and inhibiting the H_2_O_2_-induced activation of the Rho-associated kinase (ROCK) pathway [140].

Altogether, these data suggest that fucoidan may be a promising agent for the prevention/treatment of NDD. There are two main gaps still present that need to be filled. First, the function of fucoidan may vary depending on the type of algae and the extraction method. Therefore, it is seminal to perform in-depth research aiming at determining the structure of the fucoidan with the highest activity, eventually leading to the identification of the optimal source and dosage treatment, and the absorption and metabolism of this compound in vivo. Second, the need for controlled and well-designed translational studies to clarify the therapeutic efficacy of fucoidan on NDD is justified.

### 3.4. Polyphenols

Marine macroalgae, mainly brown algae, contain the highest concentrations of polyphenols, the majority of which are phlorotannins, that are unique to marine sources [141]. They are highly hydrophilic molecules containing both phenyl and phenoxy groups, that vary in structure and degree of polymerization. They are classified into four subclasses: fucols, fuhalols, fucophloroethols, and eckols [142]. Evidence from in vitro and in vivo experiments supporting the anti-inflammatory and antioxidant effects of marine polyphenols have been reviewed by Murray et al. [143]. These results indicate that these compounds exert powerful antioxidant activity by neutralizing ROS/RNS and chelating redox-active metals [144,145,146] and suppress inflammatory signaling through inhibition of NF-κB and MAPKs, while activating Nrf2/HO-1 to enhance cytoprotective pathways [126,147]. Of note, *Ecklonia cava* has shown great potential as a source of marine bioactive polyphenols, even though many data have been obtained using polyphenols extracted by others macroalgae, such as *Ishige okamurae*, *Fusu distichus*, *Alaria marginata*, *Sacharina groenlandica*, *Fusus vesiculosus*, *Sargassum marginatum*, etc [143]. Evidence of neuroprotective activities in vivo models of NDD is more limited. However, a phlorotannin-rich fraction of *Ishige foliacea* (PRFI) improved scopolamine-induced memory impairment in mice. PRFI reduced acetylcholinesterase activity in the brain, significantly decreased lipid peroxidation levels, promoting oxidative scavenging, and up-regulated the expression of BDNF, ERK, and CREB [124]. These data were confirmed by Kim et al. later on [125]. Similarly, the oral administration of *Ishige okamurae* extract (IOE) to TMT-injected mice prevented the short- and long-term memory impairments. Moreover, IOE attenuated cellular apoptosis by up-regulating the expression of BDNF, Nrf2, and HO-1 in a mouse brain [126]. Furthermore, in PD model mice, *Ecklonia cava* phlorotannins reduced dopaminergic neuronal death and improved motor outcomes (Table 1) [127]. Moreover, this treatment restored intestinal motor function and colon tissue morphology [109]. Like fucoidan, it is important to underline that further investigations are needed to identify which phlorotannin within these extracts is responsible for these effects. In line with this important issue, Ahmad et al. have recently published a study in which they could discover a potential neuroprotective compound, namely dioxinodehydroeckol, by in silico screening for the treatment and management of NDD [148].

### 3.5. Clinical Evidence and Translational Gaps for Marine-Derived Bioactive Compounds in AD, PD, MS and ALS

While preclinical evidence strongly supports the neuroprotective potential of marine-derived bioactive compounds, their real translational value is still questionable considering the scarce and indirect clinical data currently available. Therefore, before moving to the concluding remarks, we summarize here the existing clinical evidence and highlight the major gaps that still limit their application in AD, PD, MS, and ALS.

Marine-derived proteins and peptides, for example, have been tested in humans mainly in cardiometabolic settings. Trials with fish protein hydrolysates reported improvements in vascular function and blood pressure regulation, suggesting systemic anti-inflammatory and vasoprotective actions that could be relevant for neurodegenerative diseases [14]. Astaxanthin has been evaluated in several small randomized controlled trials in humans, demonstrating systemic antioxidant and anti-inflammatory activity in metabolic syndrome [149] and improvements in ocular health and visual performance [150,151]. These findings, although outside neurology, provide indirect but solid safety and dose-range references. Indeed, the therapeutic efficacy of omega-3 fatty acids have been evaluated in two clinical trials for PD treatment. A significant improvement of depressive symptoms was observed in both clinical studies, corroborating our hypothesis [152]. Marine polyphenols, particularly phlorotannins from Ecklonia cava, have been studied in small clinical trials validating pre-clinical findings, such as anti-inflammatory and antioxidative activities [143]. Across all these compound classes, the consistent gap lies in the lack of controlled trials in AD, PD, MS, and ALS cohorts, the scarcity of pharmacokinetic and bioavailability data in humans, and the heterogeneity of extraction and standardization methods. Therefore, well-designed and tailored clinical trials, integrating validated biomarkers (such as neurofilament light chain, inflammatory cytokines, or TSPO-PET imaging), should be planned and performed soon to determine the true therapeutic potential of marine bioactive compounds in NND.

## 4. Materials and Methods

The use of MeSH tool in PubMed allowed us to browse through NLM databases. Through this tool, we were able to refine our search and emphasize the relevant studies. The following search terms were combined: “neuroinflammation” AND “neurodegenerative disease” OR “Alzheimer’s disease” OR “Parkinson’s disease” OR “multiple sclerosis” OR “amyotrophic lateral sclerosis” AND “marine bioactive components” OR “bioactive-derived molecules” OR “marine-derived compounds” OR “marine-derived proteins” OR “marine-derived peptides” OR “marine-derived amino acids” OR “astaxanthin” OR “polysaccharides” OR “fucoidan” OR “polyphenols “ OR “phlorotannin” ND “mice” OR “mouse” OR “rat” OR “rodent” OR “clinical studies” OR “human studies”. The search was updated until October 2025.

## 5. Conclusions

Although diverse in structure, marine bioactive compounds share common mechanistic targets highly relevant to neurodegeneration: suppression of NF-κB and MAPKs, activation of Nrf2/HO-1, regulation of nuclear receptors (PPARgamma, SIRT1), and stabilization of mitochondrial and BBB function. These effects translate into attenuation of neurodegeneration in AD and PD, modulation of neuroinflammatory responses and demyelination in MS, and support of motor neuron survival in ALS. Notably, several compounds also act systemically, reducing oxidative lipoprotein modification, improving lipid metabolism, modulating microbiota, and dampening systemic inflammatory tone. This dual central–peripheral action provides a strong biological rationale for translational development. However, critical limitations must be acknowledged. Most of the evidence is derived from in vitro or in vivo models, and clinical validation is virtually absent. Bioavailability is often low, pharmacokinetic data are scarce, and variability in extraction and chemical characterization hampers reproducibility. Moreover, safety and tolerability in long-term human use remain insufficiently characterized.

Future directions include standardized extraction methods, optimized formulations to improve delivery (nanoparticles, liposomes), and well-designed randomized clinical trials targeting AD, PD, MS, and ALS cohorts. Combination approaches with established therapies may be particularly valuable, given the multifactorial pathogenesis of these diseases.

In summary, marine-derived bioactive molecules represent promising multi-target modulators of neuroinflammation and oxidative stress. In this perspective, the limited but informative clinical evidence, together with the clear translational gaps outlined above, underscores the urgent need for well-designed trials that integrate pharmacokinetic data, standardized formulations, and validated biomarkers in AD, PD, MS, and ALS.

Looking ahead, the integration of marine-derived bioactive compounds into the broader framework of neurodegenerative disease management may also offer novel preventive opportunities. Given their natural origin, pleiotropic biological activities, and generally favorable safety profile, these compounds could be implemented as dietary or nutraceutical adjuvants to reduce chronic low-grade inflammation and oxidative stress, two hallmarks of aging and neurodegeneration. Moreover, the convergence of marine pharmacology with precision medicine, using advanced biological systems and artificial intelligence tools, may accelerate the identification of bioactive combinations tailored to specific disease phenotypes. Such approaches will help to define not only which compounds are effective, but also for whom and under which metabolic or genetic conditions they exert the most benefit. Therefore, while marine bioactive molecules already stand out as a promising reservoir of therapeutic innovative tool, their full potential will only be realized through a multidisciplinary translational effort bridging marine biotechnology, pharmacology, and clinical neuroscience.

## Figures and Tables

**Table 1:** Effects of marine-derived components on AD, PD, MS, and ALS. Please refer to the text for definitions of all abbreviations used in the table.

Compound	Species	Models	Effects/Mode of Action	**Refs**
Peptides 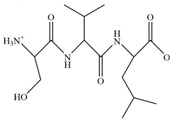	*Euphasia superba*	Scopolamine-treated mice Immunosuppressed mice	↓neuronal cell harm ↓SOD activity ↓ROS levels Immunomodulation	[99,100,101]
	*Stichopus japonicus*	Scopolamine-treated mice and rats	↓acetylcholinesterase activity ↑acetylcholine content ↑LTP pathway ↑unsaturated lipid levels	[102,103]
	*Stichopus japonicus*	D-galactose-induced aging mice	↓neuroinflammation ↑BDNF/TrkB ↓NF-kB signaling ↓gut dysbiosis ↓fecal short-chain fatty acids levels	[104]
	*Neptunea arthritica cumingii*	Zebrafish	↓locomotor impairment ↓dopaminergic neuron loss ↓loss of cerebral vessels	[105]
Astaxanthin 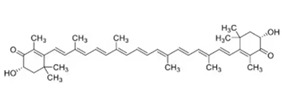	*Heamatococcus pluvialis*	SCI mice	↓post-SCI motor dysfunction ↓NF-kB signaling ↓pro-inflammatory cytokine ↑antioxidant defenses	[106]
	*Heamatococcus pluvialis*	Abeta-injected mice	↓cognitive deficits ↓GSK-3beta activity	[107]
	*Heamatococcus pluvialis*	Abeta-injected mice	↓cognitive deficits ↓GSK-3beta activity	[108]
	*Heamatococcus pluvialis*	EAE mice	↑disease prevention/progression ↓pro-inflammatory cytokine ↑Treg differentiation	[109]
	*Heamatococcus pluvialis*	MS rats	↓demyelination ↓oligodendrocyte death	[110]
Fucoidan 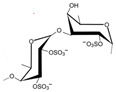	*Undaria Pinnatifida*	Abeta-injected mice	↓learning memory impairment ↑cholinergic system ↓oxidation ↓mitochondrial apoptosis ↓caspase pathway	[111]
	*Laminaria japonica*	D-galactose mice	Vlearning memory impairment ↑cholinergic system ↓oxidation ↓mitochondrial apoptosis ↓caspase pathway	[112]
Fucoidan	*Pyropia haitanensis*	Abeta-injected mice	↓learning memory impairment ↓oxidation ↓mitochondrial apoptosis	[113]
	*Ecklonia cava*	TMT-treated mice	↓cognitive dysfunctions ↓Abeta production ↓tau phosphorylation	[114]
	Brown algae	AD transgenic *C. elegans*	↓cognitive dysfunctions ↓Abeta production ↓tau phosphorylation	[115]
	*Laminaria japonica*	Rotenone-treated rats MPTP-treated mice 6-OHDA-treated rats	↓mitochondrial dysfunction by PGC-1alpha/NRF2 pathway ↑antioxidant activity ↓oxidation ↓microglial activation	[116,117,118]
	Brown algae	LPS-treated rats	↓behavioral deficits ↓microglial activation	[119]
	*Turbinaria decurrens*	MPTP-treated mice	↓behavioral deficits ↓microglial activation	[120]
	*Fucus vesiculosus*	MPTP-treated mice	↓mitochondrial dysfunction ↓motor deficits ↓neuronal apoptosis ↓dopaminergic neuron loss	[121]
	*Laminaria japonica*	Rotenone-treated mice	↓gut dysbiosis ↓permeability of the intestinal barrier ↓pro-inflammatory levels	[122]
	*--*	EAE rats	↓demyelination ↓autoreactive T cell response ↓pro-inflammatory levels	[123]
Polyphenols 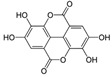	*Ishige foliacea*	Scopolamine-treated mice	↓cognitive dysfunctions ↓acetylcholinesterase activity ↓lipid peroxidation ↑ROS scavenging ↑BDNF	[124]
	*Ishige foliacea*	Scopolamine-treated mice	↓cognitive dysfunctions ↓acetylcholinesterase activity ↓lipid peroxidation ↑ROS scavenging ↑BDNF	[125]
	*Ishige okamurae*	TMT-treated mice	↓memory impairments ↓cellular apoptosis ↑BDNF ↑Nrf2 and HO-1	[126]
	*Ecklonia cava*	PD mice	↓dopaminergic neuronal death ↓motor deficits ↑intestinal motility ↑colon tissue morphology	[127]

↑: increase; ↓: reduction.

## Data Availability

No new data generated.

## References

[1] Engelhardt E., Vajkoczy P., Weller R.O. (2017). The movers and shapers in immune privilege of the CNS. Nat. Immunol..

[2] Leunig A., Gianeselli M., Russo S.J., Swirski F.K. (2025). Connection and communication between the nervous and immune systems. Nat. Rev. Immunol..

[3] Billingham R.E., Boswell T. (1953). Studies on the problem of corneal homografts. Proc. R. Soc. Lond. B Biol. Sci..

[4] Galea I., Bechmann I., Perry V.H. (2007). What is immune privilege (not)?. Trends Immunol..

[5] Louvea A., Smirnov I., Keyes T.J., Eccles J.D., Rouhani S.J., Peske J.D., Derecki N.C., Castle D., Mandell J.W., Lee K.-S. (2015). Structural and functional features of central nervous system lymphatic vessels. Nature.

[6] Smyth L.C.D., Kipnis J. (2025). Redefining CNS immune privilege. Nat. Rev. Immunol..

[7] Glass C.K., Saijo K., Winner B., Marchetto M.C., Gage F.H. (2010). Mechanisms underlying inflammation in neurodegeneration. Cell.

[8] Parolini C. (2020). Marine n-3 polyunsaturated fatty acids: Efficacy on inflammatory-based disorders. Life Sci..

[9] Yang Q., Wang G., Zhang F. (2020). Role of Peripheral Immune Cells-Mediated Inflammation on the Process of Neurodegenerative Diseases. Front. Immunol..

[10] Chiesa G., Busnelli M., Manzini S., Parolini C. (2016). Nutraceuticals and Bioactive Components from Fish for Dyslipidemia and Cardiovascular Risk Reduction. Mar. Drugs.

[11] Lamminpää I., Amedei A., Parolini C. (2024). Effects of Marine-Derived Components on Cardiovascular Disease Risk Factors and Gut Microbiota Diversity. Mar. Drugs.

[12] Parolini C. (2023). The Role of Marine n-3 Polyunsaturated Fatty Acids in Inflammatory-Based Disease: The Case of Rheumatoid Arthritis. Mar. Drugs.

[13] Amedei A., Lamminpää I., Parolini C. (2025). Potential and Future Therapeutic Applications of Eicosapentaenoic acid/Docosahexaenoic Acid and Probiotics in Chronic Low-Grade Inflammation. Biomedicines.

[14] Jensen I.-J., Maehre H.K. (2016). Preclinical and Clinical Studies on Antioxidative, Antihypertensive and Cardioprotective Effect of Marine Proteins and Peptides-A Review. Mar. Drugs.

[15] Tørris C., Småstuen M.C., Molin M. (2018). Nutrients in Fish and Possible Associations with Cardiovascular Disease Risk Factors in Metabolic Syndrome. Nutrients.

[16] Catanesi M., Caioni G., Castelli V., Benedetti E., d’Angelo M., Cimini A. (2021). Benefits under the Sea: The Role of Marine Compounds in Neurodegenerative Disorders. Mar. Drugs.

[17] Hannan M.A., Dash R., Haque M.N., Mohibbullah M., Sohag A.A.M., Rahman M.A., Uddin M.J., Alam M., Moon I.S. (2020). Neuroprotective Potentials of Marine Algae and Their Bioactive Metabolites: Pharmacological Insights and Therapeutic Advances. Mar. Drugs.

[18] Cooke J.P. (2019). Inflammation and Its Role in Regeneration and Repair. Circ. Res..

[19] Streit W.J. (2002). Microglia as neuroprotective, immunocompetent cells of the CNS. Glia.

[20] Serhan C.N. (2017). Treating inflammation and infection in the 21st century: New hints from decoding resolution mediators and mechanisms. FASEB J..

[21] Sugimoto M.A., Vago J.P., Perretti M., Teixeira M.M. (2019). Mediators of the Resolution of the Inflammatory Response. Trends Immunol..

[22] Furman D., Campisi J., Verdin E., Carrera-Bastos P., Targ S., Franceschi C., Ferrucci L., Gilroy D.W., Fasano A., Miller G.W. (2019). Chronic inflammation in the etiology of disease across the life span. Nat. Med..

[23] Gelb S., Lehtinen M.K. (2023). Snapshot: Choroid plexus brain barrier. Cell.

[24] Cui J., Xu H., Lehtinen M.K. (2021). Macrophages on the margin: Choroid plexus immune responses. Trends Neurosci..

[25] Takeuchi O., Akira S. (2010). Pattern recognition receptors and inflammation. Cell.

[26] Parolini C. (2025). Sepsis and high-density lipoproteins: Pathophysiology and potential new therapeutic targets. Biochim. Biophys. Acta Mol. Basis Dis..

[27] Angus D.C., van der Poll T. (2013). Severe sepsis and septic shock. N. Engl. J. Med..

[28] Van der Poll T., Shankar-Hari M., Wiersinga W.J. (2021). The immunology of sepsis. Immunity.

[29] Parolini C. (2025). Pathophysiology of bone remodelling cycle: Role of immune system and lipids. Biochem. Pharmacol..

[30] Sharp F.R., Bernaudin M. (2004). HIF1 and oxygen sensing in the brain. Nat. Rev. Neurosci..

[31] Manzini S., Pinna C., Busnelli M., Cinquanta P., Rigamonti E., Ganzetti S.G., Dellera F., Sala A., Calabresi L., Franceschini G. (2015). Beta2-adrenergic activity modulates vascular tone regulation in lecithin:cholesterol acyltransferase knockout mice. Vascul. Pharmacol..

[32] McGeer P.L., McGeer E.G. (2002). Inflammatory processes in amyotrophic lateral sclerosis. Muscle Nerve.

[33] McGeer P.L., McGeer E.G. (2008). Glial reactions in Parkinson’s disease. Mov. Disord..

[34] Brambilla R. (2019). The contribution of astrocytes to the neuroinflammatory response in multiple sclerosis and experimental autoimmune encephalomyelitis. Acta Neuropathol..

[35] Bayraktaroglu I., Ortí-Casañ N., Van Dam D., De Deyn P.P., Eisel U.L.M. (2025). Systemic inflammation as a central player in the initiation and development of Alzheimer’s disease. Immun. Ageing.

[36] Ertekin-Taner N. (2007). Genetics of Alzheimer’s disease: A centennial review. Neurol. Clin..

[37] Andrews S.J., Renton A.E., Fulton-Howard B., Podlesny-Drabiniok A. (2023). Marcora E, Goate AM: The complex genetic architecture of Alzheimer’s disease: Novel insights and future directions. EBioMedicine.

[38] Campion D., Dumanchin C., Hannequin D., Dubois B., Belliard S., Puel M., Thomas-Anterion C., Michon A., Martin C., Charbonnier F. (1999). Early-onset autosomal dominant Alzheimer disease: Prevalence, genetic heterogeneity, and mutation spectrum. Am. J. Hum. Genet..

[39] Ayodele T., Rogaeva E., Kurup J.T., Beecham G., Reitz C. (2021). Early-Onset Alzheimer’s Disease: What is Missing in Research?. Curr. Neurol. Nerosci. Rep..

[40] Selkoe D.J. (2011). Alzheimer’s disease. Cold Spring Harb. Perspect. Biol..

[41] Haass C., Selkoe D.J. (2007). Soluble protein oligomers in neurodegeneration: Lessons from the Alzheimer’s amyloid beta-peptide. Nat. Rev. Mol. Cell Biol..

[42] Hardy J., Selkoe D.J. (2002). The amyloid hypothesis of Alzheimer’s disease: Progress and problems on the road to therapeutics. Science.

[43] Fedele E. (2023). Anti-Amyloid Therapies for Alzheimer’s Disease and the Amyloid Cascade Hypothesis. Int. J. Mol. Sci..

[44] Behl C. (2024). In 2024, the amyloid-cascade-hypothesis still remains a working hypothesis, no less but certainly no more. Front. Aging Neurosci..

[45] Ingelsson M., Fukumoto H., Newell K.L., Growdon J.H., Hedley-whyte E.T., Frosch M.P., Albert M.S. (2004). Early Abeta accumulation and progressive synaptic loss, gliosis, and tangle formation in AD brain. Neurology.

[46] Eikelenboom P., Veerhuis R., Scheper W., Rozemuller A.J.M., van Gool W.A., Hoozemans J.J.M. (2006). The significance of neuroinflammation in understanding Alzheimer’s disease. J. Neural. Transm..

[47] Lutshumba J., Nikolajczyk B.S., Bachstetter A.D. (2021). Dysregulation of Systemic Immunity in Aging and Dementia. Front. Cell Neurosci..

[48] Akiyama H., Barger S., Barnum S., Bradt B., Bauer J., Cole G.M., Cooper N.R., Eikelenboom P., Emmerling M., Fiebich B.L. (2000). Inflammation and Alzheimer’s disease. Neurobiol. Aging.

[49] McCoy M.K., Taney M.G. (2008). TNF signaling inhibition in the CNS: Implications for normal brain function and neurodegenerative disease. J. Neuroinflamm..

[50] Elbandy M. (2002). Anti-Inflammatory Effects of Marine Bioactive Compounds and Their Potential as Functional Food Ingredients in the Prevention and Treatment of Neuroinflammatory Disorders. Molecules.

[51] Bu G. (2009). Apolipoprotein E and its receptors in Alzheimer’s disease: Pathways, pathogenesis and therapy. Nat. Rev. Neurosci..

[52] GBD 2016 Neurology Collaborators (2019). Global, regional, and national burden of neurological disorders, 1990–2016: A systematic analysis for the Global Burden of Disease Study 2016. Lancet Neurol..

[53] Braak H., Tredici D.K., Rub U., de Vos R.A.I., Steur E.N.H.J., Braak E. (2003). Staging of brain pathology related to sporadic Parkinson’s disease. Neurobiol. Aging.

[54] Perez-Pardo P., Kliest T., Dodiya H.B., Broersen L.M., Garssen J., Keshavarzian A., Kraneveld A.D. (2017). The gut-brain axis in Parkinson’s disease: Possibilities for food-based therapies. Eur. J. Pharmacol..

[55] Chen H., Zhao E.J., Zhang W., Lu Y., Liu R., Huang X., Ciesielski-Jones A.J., Justice M.A., Cousins D.S., Peddada S. (2015). Meta-analyses on prevalence of selected Parkinson’s nonmotor symptoms before and after diagnosis. Transl. Neurodegener..

[56] Gao X., Chen H., Schwarzchild M.A., Ascherio A. (2011). A prospective study of bowel movement frequency and risk of Parkinson’s disease. Am. J. Epidemiol..

[57] Abbott R.D., Petrovitch H., White L.R., Masaki K.H., Tanner C.M., Curb J.D., Grandinetti A., Blanchette P.L., Popper J.-S., Ross J.W. (2001). Frequency of bowel movements and the future risk of Parkinson’s disease. Neurology.

[58] Ponsen M.M., Stoffers D., Twisk J.W.R., Wolters E.C., Berendse H.W. (2009). Hyposmia and executive dysfunction as predictors of future Parkinson’s disease: A prospective study. Mov. Disord..

[59] Westenberger A., Skrahina V., Usnich T., Beetz C., Vollstedt E.-J., Laabs B.-H., Paul J.J., Curado F., Skobalj S., Gaber H. (2024). Relevance of genetic testing in the gene-targeted trial era: The Rostock Parkinson’s disease study. Brain.

[60] Chopra A., Lang A.E., Höglinger G., Outeiro T.F. (2024). Towards a biological diagnosis of PD. Park. Relat. Disord..

[61] Buniello A., MacArthur J.A.L., Cerezo M., Harris L.W., Hayhurst J., Malangone C., McMahon A., Morales J., Mountjoy E., Sollis E. (2019). The NHGRI-EBI GWAS Catalog of published genome-wide association studies, targeted arrays and summary statistics 2019. Nucleic. Acids Res..

[62] Marchesi M., Parolini C., Valetti C., Mangione P., Obici L., Giorgetti S., Raimondi S., Donadei S., Gregorini G., Merlini G. (2011). The intracellular quality control system down-regulates the secretion of amyloidogenic apolipoprotein A-I variants: A possible impact on the natural history of the disease. Biochim. Biophys. Acta.

[63] Gómez-Benito M., Granado N., García-Sanz P., Michel P., Dumoulin M., Moratalla R. (2020). Modeling Parkinson’s Disease with the Alpha-Synuclein Protein. Front. Pharmacol..

[64] Pauwels E.K.J., Boer G.J. (2023). Parkinson’s Disease: A Tale of Many Players. Med. Princ. Pract..

[65] Park J.-Y., Kim K.S., Lee S.-B., Ryu J.-S., Chung K.C., Choo Y.-K., Jou I., Kim J., Park S.M. (2009). On the mechanism of internalization of alpha-synuclein into microglia: Roles of ganglioside GM1 and lipid raft. J. Neurochem..

[66] Kuhn D.M., Francescutti-Verbeem D.M., Thomas D.M. (2006). Dopamine quinones activate microglia and induce a neurotoxic gene expression profile: Relationship to methamphetamine-induced nerve ending damage. Ann. N. Y. Acad. Sci..

[67] Ito M., Komai K., Mise-Omata S., Iizuka-Koga M., Nogushi Y., Kondo T., Sakai R., Matsuo K., Nakayama T., Yoshie O. (2019). Brain regulatory T cells suppress astrogliosis and potentiate neurological recovery. Nature.

[68] Saijo K., Winner B., Carson C.T., Collier J.G., Boyer L., Rosenfeld M.G., Gafe F.H., Glass C.K. (2009). A Nurr1/CoREST pathway in microglia and astrocytes protects dopaminergic neurons from inflammation-induced death. Cell.

[69] Nylander A., Hafler D.A. (2012). Multiple sclerosis. J. Clin. Investig..

[70] Sospedra M., Martin R. (2005). Immunology of multiple sclerosis. Annu. Rev. Immunol..

[71] Korn T., Bettelli E., Oukka M., Kuchroo V.K. (2009). IL-17 and Th17 Cells. Annu. Rev. Immunol..

[72] Eisen A. (2009). Amyotrophic lateral sclerosis: A 40-year personal perspective. J. Clin. Neurosci..

[73] Feldman E.L., Goutman S.A., Petri S., Mazzini L., Savelieff M.G., Saw P.J., Sobue G. (2022). Amyotrophic lateral sclerosis. Lancet.

[74] Mejzini R., Flynn L.L., Pitout I.L., Fletcher S., Wilton S.D., Akkari P.A. (2019). ALS Genetics, Mechanisms, and Therapeutics: Where Are We Now?. Front. Neurosci..

[75] Appel S.H., Zhao W., Beers D.R., Henkel J.S. (2011). The microglial-motoneuron dialogue in ALS. Acta. Myol..

[76] Chiu I.M., Morimoto E.T.A., Goodarzi H., Liao J.T., O’Keeffe S., Phatnani H.P., Muratet M., Carroll M.C., Levy S., Tavazoie S. (2013). A neurodegeneration-specific gene-expression signature of acutely isolated microglia from an amyotrophic lateral sclerosis mouse model. Cell Rep..

[77] Gravel M., Beland L.-C., Soucy G., Abdelhamid E., Rahimian R., Gravel C., Kriz J. (2016). IL-10 Controls Early Microglial Phenotypes and Disease Onset in ALS Caused by Misfolded Superoxide Dismutase 1. J. Neurosci..

[78] Beers D.R., Zhao W., Liao B., Kano O., Wang J., Huang A., Appel S.H., Henkel J.S. (2011). Neuroinflammation modulates distinct regional and temporal clinical responses in ALS mice. Brain Behav. Immun..

[79] Zhao W., Beers D.R., Liao B., Henkel J.S., Appel S.H. (2012). Regulatory T lymphocytes from ALS mice suppress microglia and effector T lymphocytes through different cytokine-mediated mechanisms. Neurobiol. Dis..

[80] Kang J., Rivest S. (2007). MyD88-deficient bone marrow cells accelerate onset and reduce survival in a mouse model of amyotrophic lateral sclerosis. J. Cell Biol..

[81] Hailman E., Lichenstein H.S., Wurfel M.M., Miller D.S., Johnson D.A., Kelley M., Busse L.A., Zukowski M.M., Wright S.D. (1994). Lipopolysaccharide (LPS)-binding protein accelerates the binding of LPS to CD14. J. Exp. Med..

[82] Sun H., Yang Y., Gu M., Li Y., Jiao Z., Lu C., Li B., Jiang Y., Jiang L., Chu F. (2022). The role of Fas-FasL-FADD signaling pathway in arsenic-mediated neuronal apoptosis in vivo and in vitro. Toxicol. Lett..

[83] Liu Y., Hao W., Dawson A., Liu S., Fassbender K. (2009). Expression of amyotrophic lateral sclerosis-linked SOD1 mutant increases the neurotoxic potential of microglia via TLR2. J. Biol. Chem..

[84] Turner M.R., Cagnin A., Turkheimer F.E., Miller C.C.J., Shaw C.E., Brooks D.J., Leigh P.N., Banati R.B. (2004). Evidence of widespread cerebral microglial activation in amyotrophic lateral sclerosis: An [11C](R)-PK11195 positron emission tomography study. Neurobiol. Dis..

[85] Nutma E., Fancy N., Weinert M., Tsartsalis S., Marzin M.C., Muirhead R.C.J., Falk I., Breur M., de Bruin J., Hollaus D. (2023). Translocator protein is a marker of activated microglia in rodent models but not human neurodegenerative diseases. Nat. Commun..

[86] Weiner H.L. (2025). Immune mechanisms and shared immune targets in neurodegenerative diseases. Nat. Rev. Neurol..

[87] McGill R.B., Steyn F.J., Ngo S.T., Thorpe Ka Heggie S., Ruitenberg M.J., Henderson R.D., McCombe P.A., Woodruff T.M. (2020). Monocytes and neutrophils are associated with clinical features in amyotrophic lateral sclerosis. Brain Commun..

[88] Yazdani S., Seitz C., Cui C., Lovik A., Piehl F., Pawitan Y., Klappe U., Press R., Samuelsson K., Yin L. (2022). T cell responses at diagnosis of amyotrophic lateral sclerosis predict disease progression. Nat. Commun..

[89] Yangou Y., Facer P., Durrenberger P., Chessell I.P., Naylor A., Bountra C., Banati R.R., Anand P. (2006). COX-2, CB2 and P2X7-immunoreactivities are increased in activated microglial cells/macrophages of multiple sclerosis and amyotrophic lateral sclerosis spinal cord. BMC Neurol..

[90] Mohd Sairazi N.S., Sirajudeen K.N.S. (2020). Natural Products and Their Bioactive Compounds: Neuroprotective Potentials against Neurodegenerative Diseases. Evid. Based Complement. Altern. Med..

[91] Jia C., Chai J., Zhang S., Sun Y., He L., Sang Z., Chen D., Zheng X. (2025). The Advancements of Marine Natural Products in the Treatment of Alzheimer’s Disease: A Study Based on Cell and Animal Experiments. Mar. Drugs.

[92] Silva J., Alves C., Soledade F., Martins A., Pinteus S., Gaspar H., Alfonso A., Pedrosa R. (2023). Marine-Derived Components: Can They Be a Potential Therapeutic Approach to Parkinson’s Disease?. Mar. Drugs.

[93] Kemp D.C., Ewon J.Y. (2021). Fish and Shellfish-Derived Anti-Inflammatory Protein Products: Properties and Mechanisms. Molecules.

[94] Ripps H., Shen W. (2012). Review: Taurine: A "very essential" amino acid. Mol. Vis..

[95] Choi J.Y., Jang J.S., Son D.J., Im H.-S., Kim J.Y., Park J.E., Choi W.R., Han S.-B., Hong J.T. (2017). Antarctic Krill Oil Diet Protects against Lipopolysaccharide-Induced Oxidative Stress, Neuroinflammation and Cognitive Impairment. Int. J. Mol. Sci..

[96] Parolini C., Bjorndal B., Busnelli M., Manzini M., Ganzetti G.S., Dellera F., Ramsvik M., Bruheim I., Berge R.F., Chiesa G. (2017). Effect of Dietary Components from Antarctic Krill on Atherosclerosis in apoE-Deficient Mice. Mol. Nutr. Food Res..

[97] Wang K., Li Y., Dai Y., Han L., Zhu Y., Xue C., Wang P., Wang J. (2019). Peptides from Antarctic Krill (*Euphausia superba*) Improve Osteoarthritis via Inhibiting HIF-2α-Mediated Death Receptor Apoptosis and Metabolism Regulation in Osteoarthritic Mice. J. Agric. Food Chem..

[98] Yue H., Cai W., Bai X., Dong P., Wang J. (2022). Antarctic krill peptide alleviates liver fibrosis via downregulating the secondary bile acid mediated NLRP3 signaling pathway. Food Funct..

[99] Zheng J., Gao Y., Ding J., Sun N., Lin S. (2022). Antarctic krill peptides improve scopolamine-induced memory impairment in mice. Food Biosci..

[100] Yang J., Qi Y., Zhu B., Lin S. (2024). A Novel Tetrapeptide Ala-Phe-Phe-Pro (AFFP) Derived from Antarctic Krill Prevents Scopolamine-Induced Memory Disorder by Balancing Lipid Metabolism of Mice Hippocampus. Nutrients.

[101] Yang T., Meng Y., Li S., Zhao X., Hou H. (2025). Preparation, unique structural characteristics and immunomodulatory effects of peptides from Antarctic krill (*Euphausia superba*). Food Res. Int..

[102] Xu X., Liang R., Li D., Jiang C., Lin S. (2020). Evaluation of sea cucumber peptides-assisted memory activity and acetylation modification in hippocampus of test mice based on scopolamine-induced experimental animal model of memory disorder. J. Funct. Foods.

[103] Lu X., Xu X., Li D., Sun N., Lin S. (2022). Sea Cucumber Peptides Attenuated the Scopolamine-Induced Memory Impairment in Mice and Rats and the Underlying Mechanism. J. Agric. Food Chem..

[104] Gong H., Zhao Mao X. (2025). Sea Cucumber Hydrolysates Alleviate Cognitive Deficits in D-Galactose-Induced C57BL/6J Aging Mice Associated with Modulation of Gut Microbiota. Foods.

[105] Ren Q., Jiang X., Zhang S., Gao X., Paudel Y.N., Zhang P., Wang R., Liu K., Jin M. (2022). Neuroprotective effect of YIAEDAER peptide against Parkinson’s disease like pathology in zebrafish. Biomed. Pharmacother..

[106] Fakhri S., Dargahi L., Abbaszadeh F., Jorjani M. (2018). Astaxanthin attenuates neuroinflammation contributed to the neuropathic pain and motor dysfunction following compression spinal cord injury. Brain Res. Bull..

[107] Rahman S.O., Panda B.P., Parvez S., Kaundal M., Hussain S., Akhtar M., Najmi A.K. (2019). Neuroprotective role of astaxanthin in hippocampal insulin resistance induced by Aβ peptides in animal model of Alzheimer’s disease. Biomed. Pharmacother..

[108] Liu N., Lyiu X., Zhang X., Zhang F., Chen Y., Li G. (2023). Astaxanthin attenuates cognitive deficits in Alzheimer’s disease models by reducing oxidative stress via the SIRT1/PGC-1α signaling pathway. Cell Biosci..

[109] Bidaran S., Ahmadi A.R., Yaghmaei P., Sanati M.H., Ebrahim-Habibi A. (2018). Astaxanthin effectiveness in preventing multiple sclerosis in animal model. Bratisl. Lek. Listy.

[110] Lofti A., Soleimani M., Ghasemi N. (2021). Astaxanthin Reduces Demyelination and Oligodendrocytes Death in A Rat Model of Multiple Sclerosis. Cell J..

[111] Wei H., Gao Z., Zheng L., Zhang C., Liu Z., Yang Y., Teng H., Hou L., Yin Y., Zou X. (2017). Protective Effects of Fucoidan on Aβ25-35 and d-Gal-Induced Neurotoxicity in PC12 Cells and d-Gal-Induced Cognitive Dysfunction in Mice. Mar. Drugs.

[112] Gao Y., Li C., Yin J., Shen J., Wang H., Wu Y., Jin H. (2012). Fucoidan, a sulfated polysaccharide from brown algae, improves cognitive impairment induced by infusion of Aβ peptide in rats. Environ. Toxicol. Pharmacol..

[113] Zhang Z., Wang X., Pan Y., Wang G., Mao G. (2020). The degraded polysaccharide from Pyropia haitanensis represses amyloid beta peptide-induced neurotoxicity and memory in vivo. Int. J. Biol. Macromol..

[114] Park S.K., Kang J.Y., Kim J.M., Yoo S.K., Han H.J., Chung D.H., Kim D.-O., Kim G.-H., Heo H.J. (2019). Fucoidan-Rich Substances from Ecklonia cava Improve Trimethyltin-Induced Cognitive Dysfunction via Down-Regulation of Amyloid β Production/Tau Hyperphosphorylation. Mar. Drugs.

[115] Wang X., Yi K., Zhao Y. (2018). Fucoidan inhibits amyloid-β-induced toxicity in transgenic Caenorhabditis elegans by reducing the accumulation of amyloid-β and decreasing the production of reactive oxygen species. Food Funct..

[116] Zhang L., Hao J., Zheng Y., Su R., Liao Y., Gong X., Liu L., Wang X. (2018). Fucoidan Protects Dopaminergic Neurons by Enhancing the Mitochondrial Function in a Rotenone-induced Rat Model of Parkinson’s Disease. Aging Dis..

[117] Luo D., Zhang Q., Wang H., Cui Y., Sun Z., Yang J., Zheng Y., Jia J., Yu F., Wang X. (2009). Fucoidan protects against dopaminergic neuron death in vivo and in vitro. Eur. J. Pharmacol..

[118] Zhang F.-L., He Y., Zheng Y., Zhang W.-J., Wang Q., Jia Y.-J., Song H.-L., An H.-T., Zhang H.-B., Qian Y.-J. (2014). Therapeutic effects of fucoidan in 6-hydroxydopamine-lesioned rat model of Parkinson’s disease: Role of NADPH oxidase-1. CNS Neurosci. Ther..

[119] Cui Y.Q., Jia Y.-J., Zhang T., Zhang Q.-B., Wang X.-M. (2012). Fucoidan protects against lipopolysaccharide-induced rat neuronal damage and inhibits the production of proinflammatory mediators in primary microglia. CNS Neurosci. Ther..

[120] Meenakshi S., Umayaparvathi S., Saravanan R., Manivasagam T., Balasubramanian T. (2016). Neuroprotective effect of fucoidan from Turbinaria decurrens in MPTP intoxicated Parkinsonic mice. Int. J. Biol. Macromol..

[121] Xing M., Li G., Liu Y., Yang L., Zhang Y., Zhang Y., Ding J., Lu M., Yu G., Hu G. (2023). Fucoidan from Fucus vesiculosus prevents the loss of dopaminergic neurons by alleviating mitochondrial dysfunction through targeting ATP5F1a. Carbohydr. Polym..

[122] Yang X., Zhang X., Ma Y., Li S., Wang Q., Hong J.-S., Yu G., Qi B., Wang J., Liu C. (2024). Fucoidan ameliorates rotenone-induced Parkinsonism in mice by regulating the microbiota-gut-brain axis. Int. J. Biol. Macromol..

[123] Kim H., Moon C., Park E., Jee Y., Ahn M., Wie M.B., Shin T. (2010). Amelioration of experimental autoimmune encephalomyelitis in Lewis rats treated with fucoidan. Phytother. Res..

[124] Um M.Y., Lim D.W., Son H.J., Cho S., Lee C. (2018). hlorotannin-rich fraction from Ishige foliacea brown seaweed prevents the scopolamine-induced memory impairment via regulation of ERK-CREB-BDNF pathway. J. Funct. Foods.

[125] Kim T.-E., Son H.J., Lim D.W., Yoon M., Lee J., Kim Y.T., Han D., Lee C., Um M.Y. (2020). Memory-enhancing effects of Ishige foliacea extract: In Vitro and in vivo study. J. Food Biochem..

[126] Kwon O.Y., Lee S.H. (2021). Ishige okamurae Suppresses Trimethyltin-Induced Neurodegeneration and Glutamate-Mediated Excitotoxicity by Regulating MAPKs/Nrf2/HO-1 Antioxidant Pathways. Antioxidants.

[127] Yasuda Y., Tokumatsu T., Ueda C., Sakai M., Sasaki Y., Norikura T., Matsui-Yuasa I., Kojima-Yuasa A. (2024). Ecklonia cava Polyphenols Have a Preventive Effect on Parkinson’s Disease through the Activation of the Nrf2-ARE Pathway. Nutrients.

[128] Houser M.C., Chang J., Factor S.A., Molho E.S., Zabetian C.P., Hill-Burns E.M., Payami H., Hertzberg V.S., Tansey M.G. (2018). Stool Immune Profiles Evince Gastrointestinal Inflammation in Parkinson’s Disease. Mov. Disord..

[129] Illiano P., Brambilla R., Parolini C. (2020). The mutual interplay of gut microbiota, diet and human disease. FEBS J..

[130] Chen Y.-F., Fan Z.-K., Gao X., Zhou F., Guo X.-F., Sinclair A.J., Li D. (2024). n-3 polyunsaturated fatty acids in phospholipid or triacylglycerol form attenuate nonalcoholic fatty liver disease via mediating cannabinoid receptor 1/adiponectin/ceramide pathway. J. Nutr. Biochem..

[131] Li K., Song X., Li H., Kuang X., Liu S., Liu R., Li D. (2024). Mussel oil is superior to fish oil in preventing atherosclerosis of ApoE-/-mice. Front. Nutr..

[132] Alugoju P., Krishna Swamy V.K.D., Anthikapalli N.V.A., Tencomnao T. (2023). Health benefits of astaxanthin against age-related diseases of multiple organs: A comprehensive review. Crit. Rev. Food Sci. Nutr..

[133] Wang C.-C., Shi H.-H., Xu J., Yanagita T., Xue C.-H., Zhang T.-T., Wang Y.-M. (2010). Docosahexaenoic acid-acylated astaxanthin ester exhibits superior performance over non-esterified astaxanthin in preventing behavioral deficits coupled with apoptosis in MPTP-induced mice with Parkinson’s disease. Food Funct..

[134] Wang L., Lu K., Lou X., Zhang S., Song W., Li R., Geng L., Cheng B. (2023). Astaxanthin ameliorates dopaminergic neuron damage in paraquat-induced SH-SY5Y cells and mouse models of Parkinson’s disease. Brain Res. Bull..

[135] Park J.S., Chyn J.H., Kim Y.K., Line L.L., Chew B.P. (2010). Astaxanthin decreased oxidative stress and inflammation and enhanced immune response in humans. Nutr. Metab..

[136] Thomas N.V., Kim S.-W. (2013). Beneficial effects of marine algal compounds in cosmeceuticals. Mar. Drugs.

[137] Holdt S.L., Kraan S. (2011). Bioactive compounds in seaweed: Functional food applications and legislation. J. Appl. Phycol..

[138] Dimitrova-Shumkovska J., Krstanoski L., Veenman L. (2020). Potential Beneficial Actions of Fucoidan in Brain and Liver Injury, Disease, and Intoxication-Potential Implication of Sirtuins. Mar. Drugs.

[139] Wang Y., Wang Q., Han X., Ma Y., Zhang Z., Zhao L., Guan F., Ma S. (2021). Fucoidan: A promising agent for brain injury and neurodegenerative disease intervention. Food Funct..

[140] Hsieh C.-H., Lu C.-H., Kuo Y.-Y., Lin G.-B., Chao C.-Y. (2019). The protective effect of non-invasive low intensity pulsed electric field and fucoidan in preventing oxidative stress-induced motor neuron death via ROCK/Akt pathway. PLoS ONE.

[141] Heffernan N., Brunton N.P., FtzGerald R.J., Smyth T.J. (2015). Profiling of the molecular weight and structural isomer abundance of macroalgae-derived phlorotannins. Mar. Drugs.

[142] Murugan A.C., Karim M.R., Yusoff M.B., Tan S.H., Asras M.F., Rashid S.S. (2015). New insights into seaweed polyphenols on glucose homeostasis. Pharm. Biol..

[143] Murray M., Dordevic A.L., Ryan L., Bonham M.P. (2018). An emerging trend in functional foods for the prevention of cardiovascular disease and diabetes: Marine algal polyphenols. Crit. Rev. Food Sci. Nutr..

[144] Kang S.-M., Cha S.-H., Ko J.-Y., Kang M.-C., Kim D., Heo S.-J., Kim J.-S., Heu M.S., Kim Y.-T., Jung W.-K. (2012). Neuroprotective effects of phlorotannins isolated from a brown alga, Ecklonia cava, against H2O2-induced oxidative stress in murine hippocampal HT22 cells. Environ. Toxicol. Pharmacol..

[145] Heo S.-J., Cha S.-H., Kim K.-N., Lee S.-H., Ahn G., Kang D.-H., Oh C., Choi Y.-U., Affan A., Kim D. (2012). Neuroprotective effect of phlorotannin isolated from Ishige okamurae against H_2_O_2_-induced oxidative stress in murine hippocampal neuronal cells, HT22. Appl. Biochem. Biotechnol..

[146] Cui Y., Amarsanaa K., Lee J.H., Rhim J.-K., Kwon J.M., Kim S.-H., Park J.M., Jung S.-C., Eun S.-Y. (2019). Neuroprotective mechanisms of dieckol against glutamate toxicity through reactive oxygen species scavenging and nuclear factor-like 2/heme oxygenase-1 pathway. Korean J. Physiol. Pharmacol..

[147] Yang Y.-I., Jung S.-H., Lee K.-T., Choi J.-H. (2014). 8,8′-Bieckol, isolated from edible brown algae, exerts its anti-inflammatory effects through inhibition of NF-κB signaling and ROS production in LPS-stimulated macrophages. Int. Immunopharmacol..

[148] Ahmad F., Sachdeva P., Sachdeva B., Singh G., Soni H., Tandon S., Rafeeq M.M., Alam Z.M., Baeissa H.M., Khalid M. (2024). Dioxinodehydroeckol: A Potential Neuroprotective Marine Compound Identified by In Silico Screening for the Treatment and Management of Multiple Brain Disorders. Mol. Biotechnol..

[149] Gao C., Gong N., Chen F., Hu S., Zhou Q., Gao X. (2024). The Effects of Astaxanthin on Metabolic Syndrome: A Comprehensive Review. Mar. Drugs.

[150] Nouchi R., Suiko T., Kimura E., Takenada H., Murakoshi M., Uchiyama A., Aono M., Kawashima R. (2020). Effects of Lutein and Astaxanthin Intake on the Improvement of Cognitive Functions among Healthy Adults: A Systematic Review of Randomized Controlled Trials. Nutrients.

[151] Hecht K.A., Marwah M., Wood V., Nishida Y., Bach A.E., Gerson J., Hom M.M., Schnackenberg J., Raote S., Srivastava S. (2025). Astaxanthin (AstaReal^®^) Improved Acute and Chronic Digital Eye Strain in Children: A Randomized Double-Blind Placebo-Controlled Trial. Adv. Ther..

[152] Li P., Song C. (2022). Potential treatment of Parkinson’s disease with omega-3 polyunsaturated fatty acids. Nutr. Neurosci..

